# Effect of sustained inflations and intermittent positive pressure ventilation on bronchopulmonary dysplasia or death among neonatal: A protocol for systematic review

**DOI:** 10.1097/MD.0000000000019084

**Published:** 2020-02-21

**Authors:** Yue Guo, Yan Jiang, Hanbo Tang, Wenjuan Fan, Chenchen Ai, Ping Liu

**Affiliations:** Gansu Provincial Maternity and Child-Care Hospital, Lanzhou, China.

**Keywords:** bronchopulmonary dysplasia, death, intermittent positive pressure ventilation, meta-analysis, mortality, preterm infants, protocol, sustained inflations

## Abstract

**Background:**

Sustained Inflations (SI) and Intermittent Positive Pressure Ventilation (IPPV) are two interventions to prevent Bronchopulmonary dysplasia (BPD). The aim of this study is to assess the effect of these two interventions.

**Methods:**

The databases of PubMed, EMBASE, and Cochrane Central Register of Controlled Trials (CENTRAL) will be comprehensively searched from inception to September 2019. All RCTs and quasi-RCTs which compare the efficacy of SI vs IPPV among preterm infants are eligible. We will assess the methodological quality using the Cochrane Handbook version 5.1.0. A meta-analysis will be performed using RevMan 5.3 software and the results will be presented using risk ratios (RRs) and 95% confidence intervals (CIs).

**Conclusions:**

This study will provide strong evidence for assessing the effect of SI and IPPV on BPD or death among preterm infants.

**PROSPERO registration number:**

CRD42019135816.

## 1 Introduction

Bronchopulmonary dysplasia (BPD), a chronic neonatal lung disease, is the most common and severe complication of preterm birth. It is well known that moderate to severe BPD affects about more than 40% preterm infants who <29 weeks’ gestational age, and the number has not changed since 2000.^[1]^ Although the neonatal nursing has been improved in the past 30 years, the BPD prevalence has not dramatically decreased.^[2]^ In recent years, BPD associated mortality has declined, but BPD is still a major mortality cause for preterm infants.^[3]^ Preterm infants with BPD may prolong hospital stays, increase healthcare costs, and risk of pulmonary morbidity.^[4–6]^ Additionally, BPD is associated with growth failure, poor school-age performance, cerebral palsy, and neurodevelopmental impairment.^[7,8]^

International consensus recommended intermittent positive pressure ventilation (IPPV), which could provide adequate tidal volume to help gas exchange and motivate breathing with lower lung injury, for infants who cannot breathe after birth.^[9,10]^ The lung of preterm infants is uniquely vulnerable to injury, so the lung of preterm infants is easily harm by mechanical ventilation.^[11]^ Studies showed that sustained inflation (SI) could increase more inspiratory volume and functional residual capacity than IPPV, and could significantly reduce the need of intubation, the incidence of BPD and intraventricular hemorrhage, and decrease the duration of hospital stay when compared with IPPV alone.^[12–14]^ SI are used frequently in Europe, and the European Resuscitation Council (ERC) also recommended 2 to 3 s SI when an infant is gasping or apneic.^[15,16]^ Well-conducted meta-analyses of RCTs are accepted as the best-quality evidence to inform clinical practice and health policy.^[17,18]^ Previous systematic reviews and meta-analyses showed that there was no benefit on the risk of mortality and BPD when compared SI with IPPV.^[19,20]^ However, this effect was not seen when the most recent trials were added. And the International Liaison Committee on Resuscitation (ILCR) concluded that more data are need.^[21]^ Kirpalani et al^[22]^ published a multicenter, high quality randomized controlled trail (RCT) with large sample size among extremely preterm infants requiring resuscitation at birth in 2019.

Thus, our study aims to assess the effect of SIs and intermittent positive pressure ventilation on BPD or death among preterm infants.

## 2 Methods

### 2.1 Protocol registration

This meta-analysis has been registered on the International Prospective Register of Systematic Reviews (PROSPERO). The registration number is: CRD42019135816. The registered title was “Effect of Sustained Inflations and Intermittent Positive Pressure Ventilation on Bronchopulmonary Dysplasia or Death Among neonatal: a protocol of meta-analysis.” Considering the clinical population heterogeneity, we decide that the present manuscript will only focus on preterm infants. Therefore, the manuscript title has been changed to “Effect of Sustained Inflations and Intermittent Positive Pressure Ventilation on Bronchopulmonary Dysplasia or Death Among Preterm Infants: a protocol of meta-analysis.”

### 2.2 Data sources and study searches

We comprehensively searched PubMed, EMBASE, and Cochrane Central Register of Controlled Trials (CENTRAL), from database inception until September 2019. The search strategies will be developed by an experienced librarian researcher to improve the search quality.^[23]^ The search terms include: infants, neonatal, newborn, and SI. There were no restrictions in terms of the year of publication or publication status.

### 2.3 Study selection

We will import initial search records into the web-application systematic review software package “Rayyan.”^[24]^ After remove duplicates, two reviewers will independently screen all titles and abstracts. Any potentially relevant studies and conflicted studies will be moved to full-text evaluation. Disagreements in full-text reviewing will be resolved through discussion or, if necessary, through adjudication by a third reviewer.

### 2.4 Eligibility criteria

RCTs and quasi-RCTs comparing the efficacy of SIs versus intermittent positive pressure ventilation among preterm infants will be included in our meta-analysis. The primary outcomes are the risk of BPD or death, the secondary outcomes including any mechanical ventilation during hospital stay; pneumothorax; intraventricular hemorrhage; and patent ductus arteriosus (i.e., needing medical treatment and/or surgical ligation). There will no restriction on the language and status of publication.

### 2.5 Data extraction

Two review authors will independently conduct data abstraction using a data extraction form developed by all the review authors.

The data extraction form contains:

1) Administrative characteristics: study author(s); year of publication; details of other relevant papers cited.
2) Trial characteristics: study design; type, duration, follow-up time; country and location of study; informed consent.
3) Participants characteristics: birth weight; gestational age; number of participants.
4) Intervention: type of ventilation device used; duration and level of pressure of sustained lung inflation (SLI).
5) Outcomes of interests. Any disagreements will be solved by discussion among review authors.

### 2.6 Assessment of risk of bias

The methodological quality of the included studies will be assessed according to the Cochrane Handbook version 5.1.0,^[25]^ which include the method of adequate sequence generation, allocation concealment, blinding of participants and personnel, incomplete outcome data, selective reporting, and other sources of bias (potential source of bias related to the specific study design used, extreme baseline imbalance, et al).

### 2.7 Data analysis

We will conduct meta-analysis using Mantel–Haenszel method in RevMan 5.3 software. For dichotomous outcomes, we will present the results as risk ratios (RRs) and 95% confidence intervals (CIs). *P*-Value < .05 will be considered as statistically significant. *I*^2^ statistic and the Q (Chi^2^) test will be used to assess heterogeneity. We will consider *I*^2^ < 25% as no heterogeneity; *I*^2^ between 25% and 49% as moderate heterogeneity; *I*^2^ > 75% as high heterogeneity. We will use fixed effect model to pool the results regardless the effect size of heterogeneity.

### 2.8 Subgroup analysis

We will conduct a subgroup analysis to examine the effect of study-level variables on the association of SIs and intermittent positive pressure ventilation on BPD or death among preterm infants. We will consider the following subgroup factors: death in the delivery room or before discharge, grade of DPB, and risk of bias.

### 2.9 Quality of evidence

Two reviewers will independently rate the quality of evidence as high, moderate, low, or very low according to the Grading of Recommendations Assessment, Development, and Evaluation (GRADE) approach.^[26]^ We will consider the quality rating of evidence for RCTs as high, but evidence will be downgraded on the basis of following reasons: study design (risk of bias), inconsistency, indirectness, imprecision, and publication bias.^[26]^

## 3 Discussion

This study will be conducted by following the Preferred Reporting Items for Systematic Review and Meta-Analysis (PRISMA) 2009 statement. The evidence of SIs and Intermittent Positive Pressure Ventilation on BPD or Death Among Preterm Infants will be well concluded in this study. The results of this study will be of great help for physicians and infants. However, the quality of include studies holds a limit for this study.

## Author contributions

**Conceptualization:** Yue Guo, Yan Jiang, Ping Liu.

**Investigation:** Yue Guo, Yan Jiang, Ping Liu, Hanbo Tang, Wenjuan Fan, Chenchen Ai.

**Methodology:** Yue Guo, Yan Jiang, Ping Liu.

**Project administration:** Yue Guo, Yan Jiang, Ping Liu.

**Supervision:** Ping Liu.

**Writing – original draft:** Yue Guo, Yan Jiang.

**Writing – review & editing:** Yue Guo, Yan Jiang, Ping Liu, Hanbo Tang, Wenjuan Fan, Chenchen Ai.
